# Host Delivered RNAi of Two Cuticle Collagen Genes, *Mi-col-1* and *Lemmi-5* Hampers Structure and Fecundity in *Meloidogyne incognita*

**DOI:** 10.3389/fpls.2017.02266

**Published:** 2018-01-22

**Authors:** Sagar Banerjee, Sarvajeet S. Gill, Bharat H. Gawade, Pradeep K. Jain, Kuppuswamy Subramaniam, Anil Sirohi

**Affiliations:** ^1^Division of Nematology, ICAR-Indian Agricultural Research Institute, New Delhi, India; ^2^Stress Physiology and Molecular Biology Lab, Centre for Biotechnology, Maharshi Dayanand University, Rohtak, India; ^3^Division of Plant Quarantine, ICAR-National Bureau of Plant Genetic Resources, New Delhi, India; ^4^ICAR-National Research Centre on Plant Biotechnology, New Delhi, India; ^5^Department of Biotechnology, Indian Institute of Technology, Chennai, India

**Keywords:** RNAi, dsRNA, siRNA, plant parasitic nematodes, *Mi-col-1*, *Lemmi-5*, cuticle collagens

## Abstract

Root-knot nematodes have emerged as devastating parasites causing substantial losses to agricultural economy worldwide. Tomato is the most favored host for major species of root-knot nematodes. Control strategies like use of nematicides have proved to be harmful to the environment. Other control methods like development of resistant cultivars and crop rotation have serious limitations. This study deals with the application of host generated RNA interference toward development of resistance against root-knot nematode *Meloidogyne incognita* in tomato. Two cuticle collagen genes viz. *Mi-col-1* and *Lemmi-5* involved in the synthesis and maintenance of the cuticle in *M. incognita* were targeted through host generated RNA interference. Expression of both *Mi-col-1* and *Lemmi-5* was found to be higher in adult females followed by egg masses and J2s. Tomato var. Pusa Ruby was transformed with the RNAi constructs of these genes to develop transgenic lines expressing the target dsRNAs. 30.80–35.00% reduction in the number of adult females, 50.06–65.73% reduction in the number of egg mass per plant and 76.47–82.59% reduction in the number of eggs per egg mass were observed for the T_1_ events expressing *Mi-col-1* dsRNA. Similarly, 34.14–38.54% reduction in the number of adult females, 62.34–66.71% reduction in number of egg mass per plant and 67.13–79.76% reduction in the number of eggs per egg mass were observed for the T_1_ generation expressing *Lemmi-5* dsRNA. The multiplication factor of *M. incognita* reduced significantly in both the cases and the structure of adult females isolated from transgenic plants were heavily distorted. This study demonstrates the role of the cuticle collagen genes *Mi-col-1* and *Lemmi-5* in the structure and development of *M. incognita* cuticle inside the host and reinforces the potential of host generated RNA interference for management of plant parasitic nematodes (PPNs).

## Introduction

Plant parasitic nematodes (PPNs) pose a major threat to world agriculture causing an estimated economic loss of around US $173 billion annually (Elling, 2013). Root knot nematodes (RKNs) are one of the most devastating PPNs with a wide host range of more than 3000 plant species. *Meloidogyne incognita* is the most predominant species of the root-knot nematodes and possibly the most damaging PPN all across the world (Trudgill and Blok, 2001). Jain et al. (2007), have attributed 27.21% losses annually in India to *M. incognita* in the solanacious crop tomato. *M. incognita* is an apomictic (mitotic parthenogenetic) and biotrophic sedentary endoparasite. Host plant roots are penetrated by infective second stage juveniles (J2s) which migrate further until they reach vascular tissues. Here, they induce the formation of multinucleate giant cells (GCs) by injecting esophageal gland secretions into root cells which become their feeding sites. The developing nematodes receive food from the feeding cells and molt thrice to form third stage juveniles (J3s), fourth stage juveniles (J4s) and adult females. Meanwhile, the cells around the developing nematode become enlarged and proliferated leading to the formation of galls (Jones and Northcote, 1972). The formation of galls interferes with upward translocation of nutrients and water in the affected roots leading to reduction in crop yield (Moens et al., 2009).

Over the years, different approaches like use of chemical nematicides, development of resistant cultivars and cultural practices have been employed for management of these devastating parasites. Chemical nematicides have been most effective as control measures for RKNs, however, use of chemical nematicides leads to serious health and environmental issues such as contamination of ground water, environmental persistence, mammalian toxicity etc. Therefore, many countries have imposed bans on major nematicides. Resistance breeding continues to be a strategy of nematode control against *Meloidogyne* spp., *Globodera rostochiensis* and *Heterodera glycines* on tomato, potato and soybean respectively (Atkinson et al., 2003); however, its success depends on the host gene pool and availability of resistant genes. This scenario indicates that the measures for the control of RKNs are currently limited. Therefore, novel approaches need to be applied for the control of RKNs. RNA interference (RNAi) is an effective tool that can be utilized to engineer resistance against RKNs. In this approach, dsRNA precursors corresponding to the target gene are processed by RNase III domain containing specialized enzymes called Dicer leading to the generation of small RNA duplexes called short interfering RNAs (siRNAs). These RNA duplexes are then incorporated into the RNA-induced silencing complex (RISC). RISC loaded with siRNA is guided to the target RNA by the core catalytic component of RISC which consists of argonoute family protein, which induces the silencing of the target gene in a sequence specific manner.

RNAi in PPNs was first demonstrated by Urwin et al. (2002) by soaking J2s of *H. glycines* and *G. palida* in dsRNA solutions. This method was later used in several studies in different PPNs (Bakhetia et al., 2005; Rosso et al., 2005; Huang et al., 2006; Dubreuil et al., 2007; Kimber et al., 2007; Shingles et al., 2007; Gleason et al., 2008; Park et al., 2008) for functional analysis of potentially lethal target genes and paved way for host generated RNAi which is a suitable approach for management of PPNs owing to their obligate parasitic nature. The target genes for host generated RNAi are selected on the basis of their involvement in the various stages of nematode development, parasitism, reproduction etc. Host generated RNAi was first demonstrated by Yadav et al. (2006) in tobacco against *M. incognita* by targeting two housekeeping genes, integrase and splicing factor. Since then numerous studies have reported host generated RNAi against RKNs targeting various housekeeping genes, parasitism genes and genes associated with nematode development (Huang et al., 2006; Fairbairn et al., 2007; Niu et al., 2012, 2016; Antonino de Souza Júnior et al., 2013; Papolu et al., 2013; Xue et al., 2013; Dinh et al., 2014a,b; Dutta et al., 2015; Lourenço-Tessutti et al., 2015; Zhuo et al., 2016; Kumar et al., 2017) with varying degree of success toward achieving resistance in *Arabidopsis thaliana*, tobacco, tomato and potato.

Cuticle collagens are the key components of RKN cuticle which maintains the shape of the nematode, protects it from external environment and plays important role in its interaction with the host and soil environment (Davies and Curtis, 2011). Cuticle collagen genes have been well explored in the model free living nematode *Caenorhabditis elegans* (Johnstone, 2000), however, these are still understudied in PPNs. *M. incognita* molts multiple times throughout its life cycle and needs to form new cuticle during each molting process. Cuticle collagen genes of PPNs have not been targeted till date for host generated RNAi. However, they can be effective targets for silencing owing to their involvement in the development of the nematodes during their parasitic life cycle. In this study, we have targeted two cuticle collagen genes of *M. incognita*; *Mi-col-1* and *Lemmi-5* for host generated RNAi in tomato to assess its effect on the development and parasitism of the nematode.

## Materials and methods

### Nematode culture and its maintenance

*Meloidogyne incognita* Chitwood race I was maintained on young tomato plants (*Solanum lycopersicum* L. cv. Pusa Ruby) inoculated with 2 J2s/gram of soil. Infected plants were uprooted 30 days post inoculations and egg masses were handpicked after washing the roots with double distilled water. The egg masses were kept in a cavity block and surface sterilized with 0.1% HgCl_2_ for 1 min. The surface sterilizing agent was then removed by washing the egg masses thrice with double distilled water. The egg masses were allowed to hatch at 26–28°C through a wire gauge covered with double layered tissue paper into a petri plate filled with double distilled water (Hooper, 1986). Freshly hatched J2s were used for further experiments. Adult females were also isolated from the roots of the infected tomato plants 30 days post inoculation under the microscope using a needle.

### Target genes for silencing and their *in silico* characterization

Cuticle collagen genes, *Mi-col-1* and *Lemmi-5* were selected for silencing through host delivered RNAi to demonstrate the effect of downregulation of these structural genes in the parasitic life cycle of *M. incognita*. The nucleotide sequences of both the genes and their corresponding amino acid sequences were retrieved from NCBI database. Domains were predicted from the amino acid sequences of these genes using SMART (Letunic et al., 2015). A phylogenetic tree was constructed using the amino acid sequences of the cuticle collagens of the plant parasitic and free living nematodes available in the database using MEGA6 (Tamura et al., 2013). TMHMM 2.0 server was used for prediction of transmembrane domains.

### Cloning and sequencing of *Mi-col-1* and *Lemmi-5*

Total RNA was extracted from adult females of *M. incognita* using TRIzol reagent (Invitrogen) following manufacturer's protocol followed by DNase I (Thermo Fischer Scientific) treatment. The total RNA was quantified using Nanodrop spectrophotometer (Thermo Fischer Scientific) and first strand cDNA was synthesized with 1 μg of the total RNA using PrimeScript™ first strand cDNA synthesis kit (Takara). 416 and 799 bp long fragments of *Lemmi-5* and *Mi-col-1*, respectively were amplified using gene specific primers (Table 1). The amplified products were run on 1% agarose gel and subsequently eluted by using GeneJET gel extraction kit (Thermo Fischer Scientific). The eluted products were cloned into pGEMT easy vector (Promega) following manufacturer's protocol. The ligated products were transformed into *E. coli* DH5α and positive colonies were screened through blue white selection and colony PCR. Plasmids were isolated from PCR positive colonies using GeneJET plasmid miniprep kit (Thermo Fischer Scientific) following manufacturer's protocol and confirmed for the presence of the inserts by restriction digestion by *Eco* RI. The inserts in the plasmid were custom sequenced (ABI SOLiD sequencing system) to reassert their identity.

**Table 1:** List of primers used for PCR amplification, pGEMT cloning and southern blotting, RNAi vector cloning and expression analysis.

**Gene**	**Primer name**	**Primer sequence**
**PCR AMPLIFICATION, pGEMT CLONING AND SOUTHERN BLOTTING**		
*Mi-col-1*	col-1-F	TACGAGGCCAGAATTAAGGC
	col-1-R	CATTTCCAGGTTGACCGGGT
*Lemmi-5*	Lemmi-5-416-F	AGCCTCGTTCTAACCCCTTC
	Lemmi-5-416-R	TCCGTTCTTTCCTGGTTGTC
**RNAi VECTOR CLONING**		
*Lemmi-5-attb*	Lemmi-5-attbF	GGGGACAAGTTTGTACAAAAAAGCAGGCTAGCCTCG
		TTCTAACCCCTTC
	Lemmi-5-attbR	GGGGACCACTTTGTACAAGAAAGCTGGGTTCCGTTC
		TTTCCTGGTTGTC
*Mi-col-1*	Mi-col-1-SF	TCTGCAGGATCCTACGAGGCCAGAATTAAGGC
	Mi-col-1-SR	TCTGCACTCGAGCATTTCCAGGTTGACCGGGT
	Mi-col-1-ASF	TCTGCAGAGCTCTACGAGGCCAGAATTAAGGC
	Mi-col-1-ASR	TCTGCAGGTACCCATTTCCAGGTTGACCGGGT
**EXPRESSION ANALYSIS**		
*Mi-col-1*	Col-1-qrt-F	TCAACCTGGAAATGACGGAG
	Col-1-qrt-R	CGTCGATGGCGCAATATTTG
*Lemmi-5*	Lemmi-5-qrt-F	CTCTGCTTGGAATGATTTGATGG
	Lemmi-5-qrt-R	GGAACGTTTCTGCCTGTAGAG
*18S rRNA (M. incognita)*	18S-Mi-qrt-F	TCAACGTGCTTGTCCTACCCTGAA
	18S-Mi-qrt-R	TGTGTACAAAGGGCAGGGACGTAA
*18S rRNA (S. lycopersicum)*	18S-Sl-qrt-F	CGCGCGCTACACTGATGTATTCAA
	18S-Sl-qrt-R	TACAAAGGGCAGGGACGTAGTCAA

### Differential expression of *Mi-col-1* and *Lemmi-5* at different life stages of *M. incognita*

Expression of *Mi-col-1* and *Lemmi-5* was quantified at different life stages of *M. incognita* through quantitative real time PCR (qRT-PCR) using cDNAs from J2s, egg masses and adult females. Total RNA was isolated and from these developmental stages, quantified and first stranded cDNA was synthesized as described above. A constitutively expressed gene, *18s rRNA* was used as an internal control to normalize the gene expression levels. qRT-PCR was performed in a CFX96 real time system (Biorad) using 2X brilliant III SYBR Green q-PCR master mix (Agilent). 20 μL reaction mixture for each sample consisted of 10 μL of 2X SYBR green qPCR master mix, 500 nM of each gene specific primer (Table 1) and 100 ng of first strand cDNA. Two biological and three technical replicates were taken for each sample. The amplification reactions included initial denaturation of 95°C for 5 min, followed by 35 cycles of 95°C for 30 s and 60°C for 1 min. A melt curve analysis at 60–95°C was performed after 35 cycles for assessment of the amplification specificity. Fold change in gene expression was quantified by 2^−ΔΔCT^ method using mean Ct values for each amplification (Livak and Schmittgen, 2001).

### Development of RNAi constructs for *Mi-col-1* and *Lemmi-5*

Partial sequences of *Mi-col-1* (799 bp) and *Lemmi-5* (416 bp) were amplified from their respective pGEMT clones. *Lemmi-5* fragment was further cloned into pDONR 221 entry vector using attB1 and attB2 sites at the upstream and downstream of the gene sequence, respectively. Primer details are given in Table 1. pHELLSGATE12 [Obtained from Dr. Tushar Kanti Dutta, Division of Nematology, Indian Agricultural Research Institute, New Delhi] was used as a destination binary vector for development of the *Lemmi-5* RNAi construct through recombination based gateway cloning technology mediated by LR recombination reaction catalyzed by LR clonase enzyme (Invitrogen). *Mi-col-1* RNAi construct was developed using conventional cloning method with the binary vector pBC-6 (Yadav et al., 2006). The sense strand for *Mi-col-1* was cloned with *Bam*HI and *Xho*I while the antisense strand was cloned with *Kpn*I and *Sac*I restriction enzymes. The recombinant clones for both the genes were transformed to *E. coli* DH5α cells. Presence of the inserts was confirmed by colony PCR and restriction digestion. Binary plasmids containing the RNAi constructs of *Lemmi-5* and *Mi-col-1* were independently transformed into *Agrobacterium tumefaciens* EHA 105 using freeze and thaw method (Jyothishwaran et al., 2007). Selection of positive clones was done through colony PCR and restriction digestion. The positive clones were maintained in yeast extract peptone medium supplemented with selection antibiotic rifampicin (25 μg mL^−1^). In addition, spectinomycin (100 μg mL^−1^) was used for the selection of pHELLSGATE12 clones while kanamycin (100 μg mL^−1^) was used for the selection of pBC-6 clones.

### Mobilization of the RNAi constructs in tomato through *Agrobacterium* mediated transformation

Tomato (cv. Pusa Ruby) seeds were treated with tween 20 for 15 min followed by 3–4 washes with double distilled water. The seeds were treated 70% ethanol for 1 min and washed with autoclaved double distilled water for 3–4 times. These seeds were further sterilized with 4% NaOCl for 8–10 min followed by 4–5 washes with autoclaved double distilled water. Sterile seeds were germinated on half strength Murashige and Skoog (MS) agar medium (pH 5.8). Cotyledonary leaves taken from 12 to 15 days old tomato seedlings were pre-cultured in Petri dishes containing pre-cultivation media (MS + 0.5 mg L^−1^ IAA + 1 mg L^−1^ zeatin) with their abaxial surface in contact with the medium at 25°C for 2 days. Healthy explants after pre-culturing were infected with *A. tumefaciens* EHA105 harboring the RNAi constructs for 15 min with gentle shaking. These explants were blotted on sterile filter paper and co-cultured on the same pre-cultivation medium for 48 h at 28°C with 20–25 explants in each 9 cm Petri plate. After 48 h of co-cultivation under dark conditions, the co-cultivated explants were washed with 250 mg/L cefotaxime for 30 min and blotted on sterile filter paper and transferred to selection medium (MS + 0.5 mg L^−1^ IAA + 1 mg L^−1^ zeatin + 100 mg L^−1^ kanamycin + 250 mg L^−1^cefotaxime). The selection plates were incubated at 24°C with 16/8 h of light/dark photoperiod. Healthy calluses were cut and transferred to shooting medium (MS + 0.5 mg L^−1^ IAA + 2 mg L^−1^ zeatin + 100 mg L^−1^ kanamycin + 250 mg L^−1^ cefotaxime) for shoot induction and elongation. After every 15 days, the healthy explants were sub-cultured into fresh shooting medium for shoot induction and elongation. The regenerated shoots were excised from the callus and transferred on to the rooting medium (MS + 0.5 mg L^−1^ IAA + 100 mg L^−1^ kanamycin + 250 mg L^−1^cefotaxime). After 20–25 days, tomato plantlets with well-developed shoots and roots were transferred to 10 cm diameter pots containing 50% soil rite mixed with autoclaved soil for hardening. After hardening, the plants were transferred to growth chambers and maintained under the controlled condition at 25 ± 2°C with a photoperiod of 16/8 h (light/dark) at National Phytotron Facility, ICAR-IARI, New Delhi.

### Molecular confirmation of T_0_ transgenic events by PCR

Healthy leaves from T_0_ events were used for genomic DNA isolation from cetyltrimethyl-ammonium bromide (CTAB) method (Murray and Thompson, 1980). Approximately one gram of fresh, green and healthy leaf tissues were ground to fine powder in pre-chilled mortar and pestle using liquid nitrogen. 15 mL of pre-warmed (65°C) CTAB-DNA extraction buffer (1 M Tris-HCl; 0.5 M EDTA pH 8.0; 4 M NaCl and 0.1 M β-mercaptoethanol) was added to each powdered leaf sample and each sample was transferred to autoclaved Oakridge centrifuge tubes. These tubes containing the samples were incubated at 65°C in the water bath for an hour with intermittent mixing. 15 mL of phenol: chloroform: isoamyl alcohol (25:24:1) was added to each sample and mixed gently by inverting the tubes followed by centrifugation at 20,000 × g for 15 min at room temperature. The upper aqueous layer from the tubes were carefully pipetted out and transferred into new Oakridge tubes. 12 mL of chloroform: isoamyl alcohol (24:1) mixture was added to each tube containing the aqueous layer obtained from previous step followed by centrifugation at 20,000 × g for 15 min at room temperature. The upper aqueous layer from the tubes were carefully pipetted out and transferred into new Oakridge tubes and 0.6 volume of chilled isopropanol was added to each tube and stored overnight at −20°C for precipitation. After overnight incubation, the tubes were centrifuged at 10,000 × g for 10 min at 4°C. The supernatant from each tube was discarded and pellets were washed with 2 mL of 70% ethanol by centrifugation at 10,000 × g for 10 min at 4°C. The supernatant from each tube was discarded completely and the pellets were allowed to air-dry. Each pellet sample was dissolved in 200 μl of nuclease free water. For purification of DNA, 1 μl of RNase A (10 mg/ml stock) was added to each tube and incubated at 37°C for 2 h in a dry bath. Equal volumes of phenol: chloroform: isoamyl alcohol in the ratio of 25:24:1 was added to each tube, mixed by inversion and centrifuged at 10,000 × g for 5 min at room temperature. The upper aqueous phase was collected in fresh microcentrifuge tubes, 2 volumes of chilled isopropanol was added to each tube, mixed properly by inverting and incubated overnight at −20°C. Next day, the tubes were subjected to centrifugation at 10,000 × g for 5 min at 4°C and each pellet was washed with 2 ml of 70% ethanol, air dried and dissolved in 100 μl of nuclease free water. Purity of the DNA was checked on agarose gel (0.8%) and quantification was done using nanodrop spectrophotometer (Thermo Fischer Scientific).

The presence of the transgenes in the putative T_0_ plants was confirmed through PCR using gene specific primers (Table 1). Each PCR mixture (25 μl) contained 100 ng of first strand cDNA, 1 × Taq buffer, 10 mmol/L dNTP, 20 μmol/L of each primer, 3.5 mmol/L MgCl2 and 1.5 U Taq DNA polymerase (Fermentas). The PCR products were separated on 1.2% agarose gel through gel electrophoresis.

### Development of T_0_ clones and T_1_ transgenic events

Clones of T_0_ tomato plants were developed through hydroponics. Growing branches from T_0_ plants were cut appropriately and put on conical flasks filled with water at the National Phytotron Facility. T_0_ clones with well-developed roots were transferred to 10 cm pots after the development of healthy roots. Fruits were harvested from T_0_ tomato plants, seeds were extracted, germinated and the developing seedlings were transferred to 10 cm pots.

### Molecular analysis of T_1_ transgenic tomato events

#### DNA isolation from T_1_ transgenic tomato events and PCR

Genomic DNA was isolated and purified as described previously from putative T_1_ tomato plants. The presence of the transgenes in the putative T_1_ plants was confirmed through PCR using gene specific primers for *Mi-col-1* and *Lemmi-5* as described previously.

#### Southern blot to confirm the transgene integrity

Twenty microgram of genomic DNA isolated from T_1_ tomato transgenic plants were used for southern blotting through non-radioactive biotin labeling method. The genomic DNA of each transgenic event and control was digested with 50 units of *Bam*HI and *Hind*III restriction enzymes in the recommended 1X NEB buffer (New England Biolabs) separately. The 50 μL reaction mixtures were incubated at 37°C overnight. The digested DNA was separated on 0.8% agarose gel. The gel was pre-run at 30 V for 10 min followed by loading of sample in the gel. The voltage applied across the gel was 90 V for 4–5 h. The gel was stained with ethidium bromide (30 μL/300 mL) for 15 min and visualized under UV light. The gel was then soaked in gel tray containing 250 mL of de-purination solution for 15 min. Subsequently, the gel was washed with single distilled water three times and soaked in 250 mL of denaturation solution with gentle shaking for 45 min on rocker platform followed by four times washing with sterile distilled water and again soaked in 250 mL sterile neutralization buffer with gentle shaking for 15 min on a rocker platform.

The assembly for dry capillary blotting was set up. Whatman paper (No. 3) wick was kept on platform in such a way that the ends dipped well in the 10X SSC. 10X SSC was poured on the wick and bubbles were removed by rolling a clean glass rod over it. The gel was carefully kept inverted on the wick. A positively charged nylon membrane (Axiva) was cut to the same size as the gel, soaked in 10X SSC for 10 min and placed over the gel. Three pieces of Whatman paper (No. 3) of the same size as the gel were soaked in 10X SSC and placed over the membrane. A clean glass rod was rolled over to remove the excess of SSC buffer and air bubbles which may interfere with the transfer process. On this, 5–7 cm thick stack of dry blotting sheets (of same size as the gel) were placed, followed by a glass-plate and a weight of 500 g. The transfer was allowed to continue for 18 h, after which the assembly was dismantled and membrane was washed with 2X SSC buffer. The DNA transferred onto the membrane was cross-linked by exposing the membrane to UV rays (120 mJ) for 90 s and the blot was stored at 4°C for further use. The probes were prepared using biotin decalabel DNA labeling kit (Thermo Fischer Scientific) according to manufacturer's protocol. The membranes were treated with the prehybridization solution for 3–4 h with shaking at 42°C followed by subsequent treatment with hybridization solution overnight at 42°C. The presence of the transgenes was detected using biotin chromogenic detection kit using manufacturer's protocol (Thermo Fischer Scientific).

#### Quantitative expression of *Mi-col-1* and *Lemmi-5* dsRNAs in transgenic lines

To quantify the expression of *Mi-col-1* and *Lemmi-5* in the transgenic tomato events, leaves were cut from the PCR positive T_1_ plants and total RNA was isolated, quantified and first strand cDNA were synthesized. For analyzing the expression of the target genes, qRT-PCR was performed as described above. *18sRNA* gene (*S. lycopersicum*) was used as an internal control. The primer details are mentioned in Table 1.

#### Nematode infection assays for transgenic plants expressing *Mi-col-1* and *Lemmi-5* dsRNAs

T_1_ transgenic plants were grown in 10 cm pots in a growth chamber at 24°C with 16/8 h light/dark photoperiod. 20 days old T_1_ and untransformed control plants were inoculated with approximately 1000 freshly hatched J2s and were grown at National Phytotron Facility, New Delhi at 24°C with 16/8 h light/dark photoperiod for 35 days. After 35 days, the plants were uprooted, the roots were washed, and total number of egg masses, eggs per egg mass, galls and adult females were counted for each individual plant. The size and shape of the adult females were observed under the microscope (Eclipse 80i and stereomicroscope SMZ1000, Tokyo, Japan) and images were captured using Nikon DS-Fi2 camera attached to the microscope. The area and diameter of the females isolated from wild type and transgenic plants were measured using the microscope software. Nematode multiplication factor [(number of egg masses × number of eggs per egg mass) ÷ nematode inoculum level] was also calculated for each plant to assess the effect of host generated RNAi on reproductive potential of *M. incognita*. Sets of data obtained for T_1_ transgenic plants expressing *Mi-col-1* and *Lemmi-5* dsRNAs were compared with that of wild type untransformed plants. Six replicates per treatment were used for this study and the data sets were subjected to one-way analysis of variance (ANOVA). The means were reported as significant or non-significant according to the Tukey's test (*P* < 0.05) using SPSS statistics software (Version-2.0, IBM, Chicago, USA).

#### Target gene expression analysis in adult females extracted from transgenic plants

Adult females were extracted from untransformed wild type and T_1_ transgenic plants expressing *Mi-col-1* and *Lemmi-5* dsRNAs 35 days post inoculation. Total RNA was isolated from these adult females and first strand cDNA was synthesized as described above. Transcript abundance of *Mi-col-1* and *Lemmi-5* was quantified using real time PCR as described above. *M. incognita 18s rRNA* was used as internal control gene. Primer details are given in Table 1. Two biological and three technical replicates were used for this study.

## Results

### *In silico* analysis of *Mi-col-1* and *Lemmi-5*

**Figure 1:**
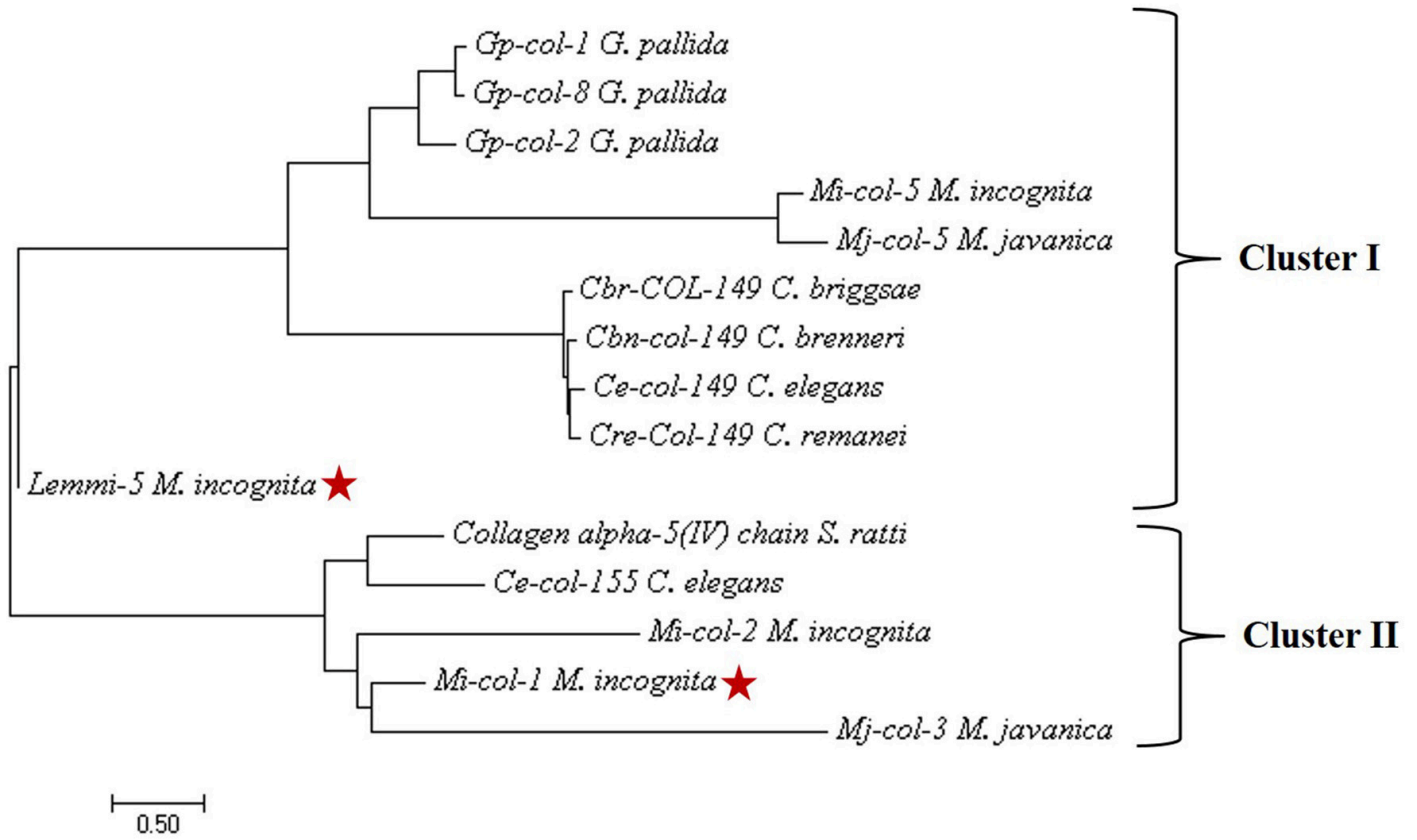
Phylogenetic tree constructed by maximum likelihood method using MEGA 7 showing evolutionary relationship of *Mi-col-1 and Lemmi-5* with cuticle collagens of other plant parasitic, animal parasitic and free living nematodes.

Nucleotide sequences of *Mi-col-1* (Accession no. U40766) and *Lemmi-5* (Accession no. AF006727) were retrieved from NCBI Genbank. The nucleotide sequences of these two genes showed no significant similarities. Further, the conserved cystein patterns of the deduced amino acid sequences revealed that *Mi-col-1* belongs to group 3 of nematode cuticle collagens, while *Lemmi-5* belongs to group 1a according to the classification proposed by Johnstone (2000) (Figure S1). Primary structural properties of the deduced amino acid sequences are listed in Table S1. SMART domain analysis revealed the presence of nematode cuticle collagen N terminal domains in *Mi-col-1* (between amino acids 12–64), as well as *Lemmi-5* (between amino acids 5–58). In *Mi-col-1*, two pfam collagen domain between amino acids 160–219 and 220–219 were also predicted (Figure S2). Presence of a transmembrane domain was predicted for both the sequences (Figure S3), however, amino acid composition in the domain varied for *Mi-col-1* and *Lemmi-5*. Phylogenetic tree based on predicted amino acid sequences showed two clusters. *Lemmi-5* formed a separate sub-cluster within cluster I, while *Mi-col-1* was placed in cluster II and showed close relationship with *Mi-col-2* and *Mj-col-3* (Figure 1).

### Differential expression of *Mi-col-1* and *Lemmi-5* at different life stages of *M. incognita*

**Figure 2:**
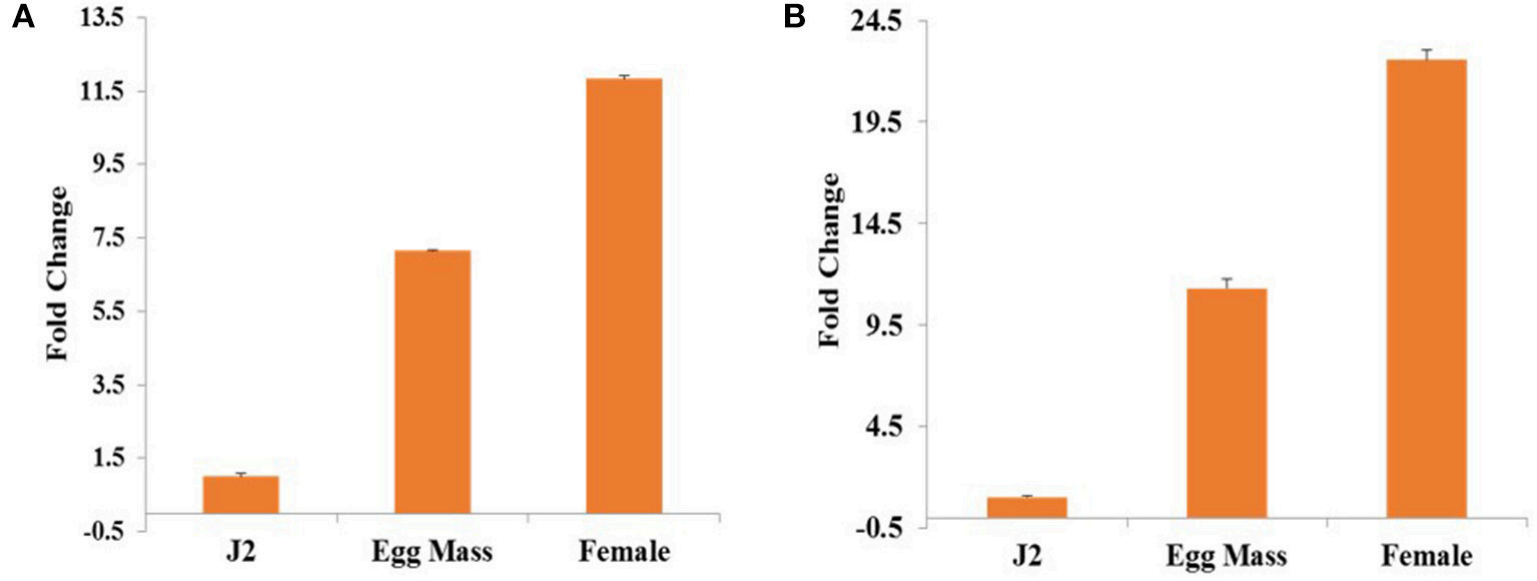
Differential expression of **(A)** *Mi-col-1* and **(B)** *Lemmi-5* in different life stages of *M. incognita* through qRT-PCR. *18s rRNA* (*M. incognita*) gene has been used as an internal control. Each bar represents the mean ± SE of *n* = 3.

Relative expression of *Mi-col-1* and *Lemmi-5* in egg masses, J2s and adult female stages of *M. incognita* was quantified through qRT-PCR. Expression of both the genes were found to be maximum in adult females followed by egg masses and J2s. Using expression levels in pre parasitic J2s as reference, 11.8 folds higher expression of *Mi-col-1* was observed in adult females, while around 7 folds higher expression of *Mi-col-1* was found in egg masses. Similarly, expression of *Lemmi-5* was around 22.5 and 11.3 folds higher in adult females and egg masses, respectively compared to its expression in pre parasitic J2s (Figure 2).

### Transformation of tomato plants and their molecular analysis for T-DNA integration

**Figure 3:**
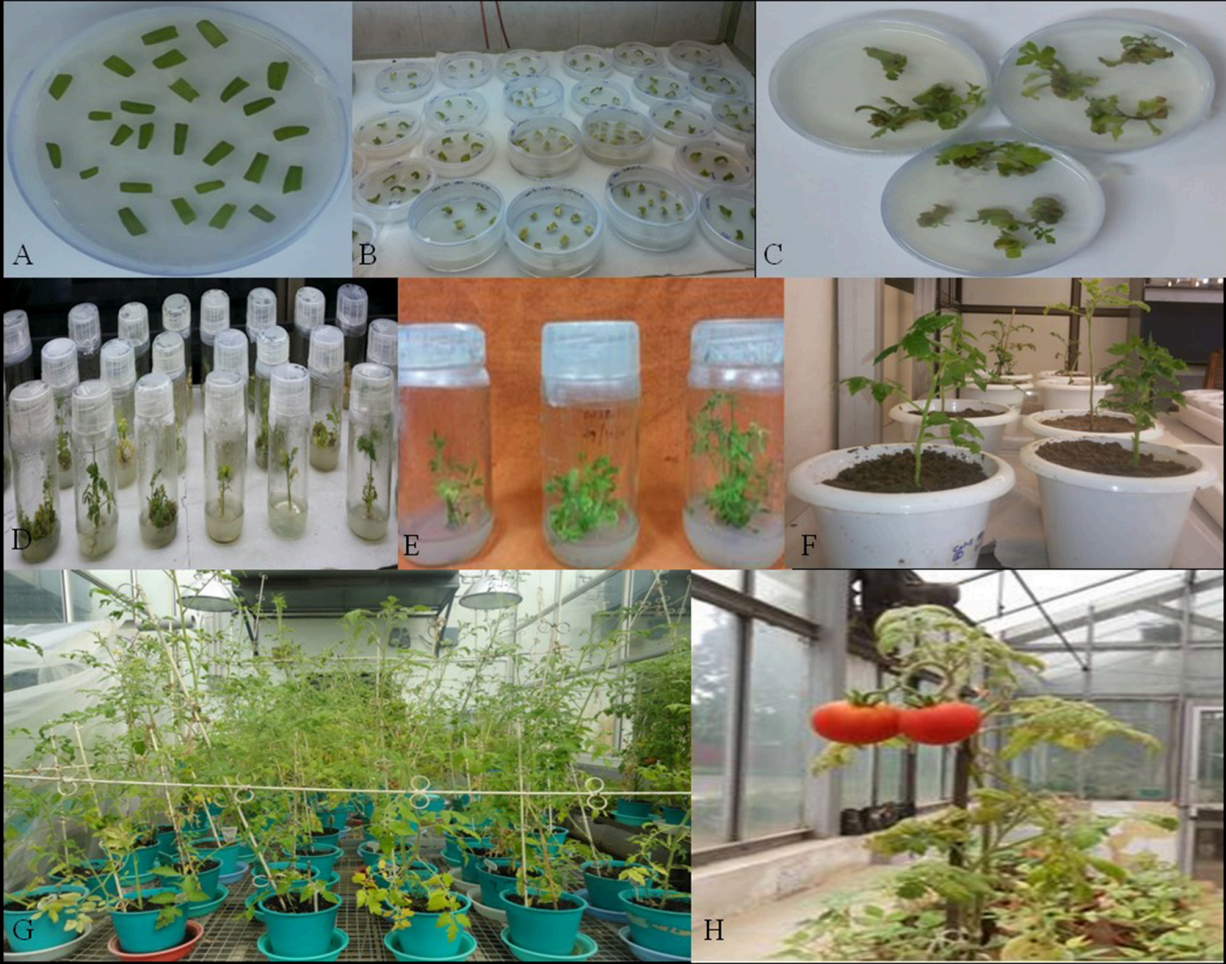
Different stages in the development in transgenic tomato plants carrying *Mi-col-1* and *Lemmi-5* dsRNAs independently. **(A)** Tomato leaf explants in the precultivation medium, **(B)** Explants in the selection medium, **(C)** Development of callus in the selection medium, **(D)** Development of shoots in the shooting medium, **(E)** Development of roots in the rooting medium, **(F)** Putative transgenics in the pots for hardening, **(G)** Further growth and hardening of putative transgenic plants in the National Phytotron Facility, **(H)** Development of fruits in the putative transgenic plants.

**Figure 4:**
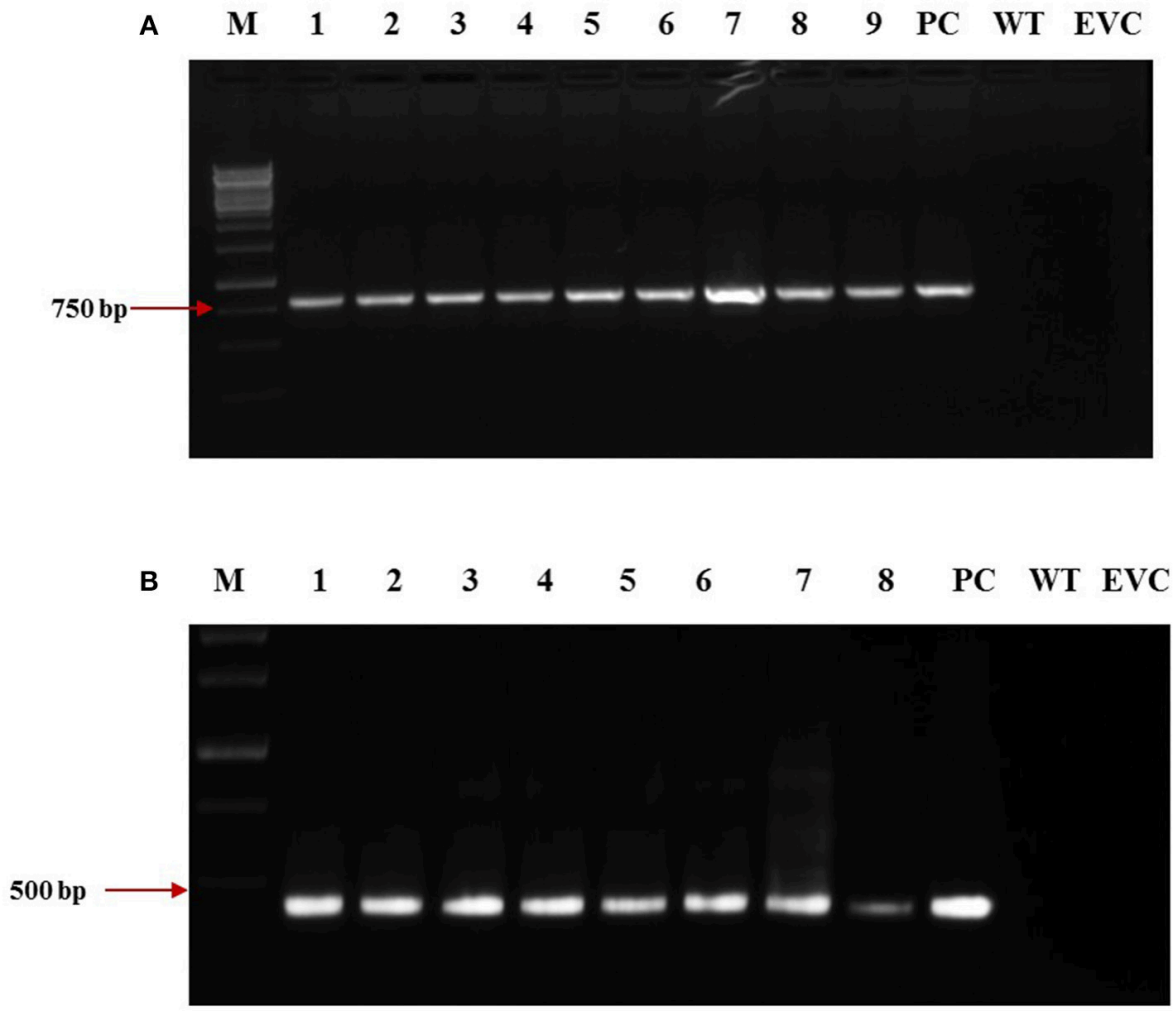
Molecular confirmation of T_0_ transgenic events for the presence of the transgene through PCR using gene specific primers. **(A)** Amplification of *Mi-col-1* insert from the DNA isolated from putatively transgenic events of *Mi-col-1*. Lane M: 1 kb ladder, lanes 1–9: T_0_ transgenic events, lane PC: positive control (pGEMT clone of *Mi-col-1*), lane WT, Wild type; lane EVC, Empty vector control; **(B)** Amplification of *Lemmi-5* insert from the DNA isolated from putatively transgenic events of *Lemmi-5*. Lane M: 1 kb ladder, lanes 1–8: T_0_ transgenic events, lane PC, positive control (pGEMT clone of *Lemmi-5*); lane WT, Wild type; lane EVC, Empty vector control.

dsRNA constructs of *Mi-col-1* and *Lemmi-5* were transformed into tomato plants through *A. tumifaciens* mediated transformation to generate T_0_ population (Figure 3). Preliminary screening of T_0_ events was carried out by PCR with target gene specific primers. Amplification of 799 bp gene specific DNA of *Mi-col-1* was observed in all the nine T_0_ events of *Mi-col-1*. Similarly, 416 bp long fragment was amplified from all the eight putative events of *Lemmi-5* (Figure 4).

### Molecular analysis of T_1_ transgenic plants through PCR and southern blotting

**Figure 5:**
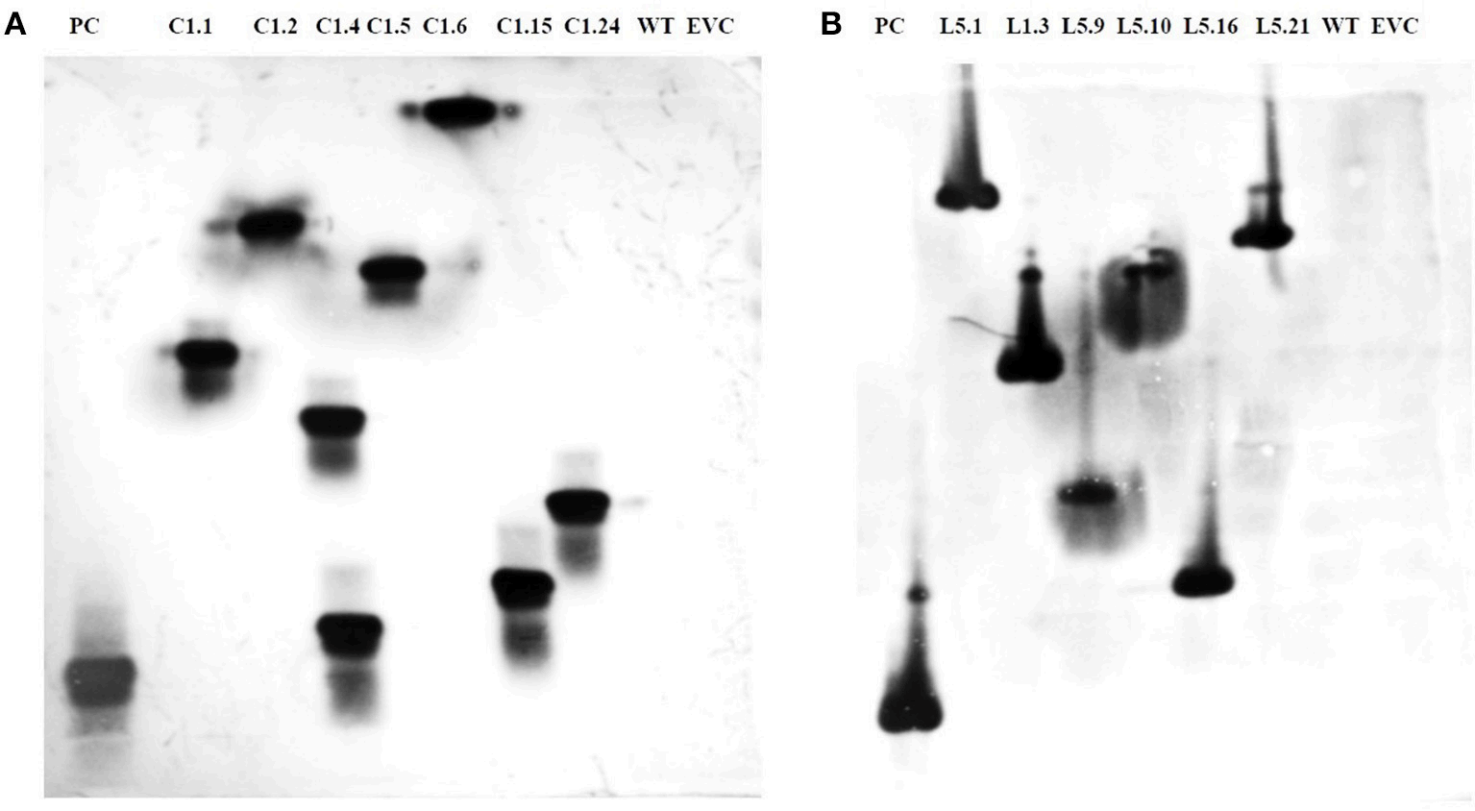
Southern blot depicting T-DNA integration in T_1_ plants harboring dsRNA of **(A)** *Mi-col-1* and **(B)** *Lemmi-5. Mi-col-1*events are C1.1, C1.2, C1.4, C1.5, C1.6, C1.15 and C1.24; *Lemmi-5* events are L1.1, L1.3, L1.9, L1.10, L1.16, and L1.21; PC denotes positive control probe of respective genes; WT denotes untransformed wild type tomato “Pusa Ruby,” EVC denotes empty vector controls for the respective gene constructs.

T_1_ plants were generated from the seeds obtained from T_0_ plants grown in the NPF, ICAR-IARI, New Delhi. A total of 7 T_1_ events were generated for *Mi-col-1*, while 6 events were generated for *Lemmi-5*. Preliminary screening of T_1_ plants was carried out by PCR with target gene specific primers. Genomic DNA was isolated from leaf tissues of putative T_1_ plants of *Mi-col-1* and *Lemmi-5*. Presence of transgene was detected in all the T_1_ events for *Mi-col-1* and *Lemmi-5* RNAi transgenic lines. The PCR confirmed T_1_ events for *Mi-col-1* and *Lemmi-5* were further subjected to southern blotting for T-DNA integration and copy number analysis. Out of the seven *Mi-col-1* transgenic events, C1.4, had two insertions, while all other events had single insertions. All the six transgenic events for *Lemmi-5* had single insertions. Hybridization bands were not detected in wild type untransformed and empty vector controls (Figure 5).

### Quantitative expression of *Mi-col-1* and *Lemmi-5* in T_1_ transgenic events

**Figure 6:**
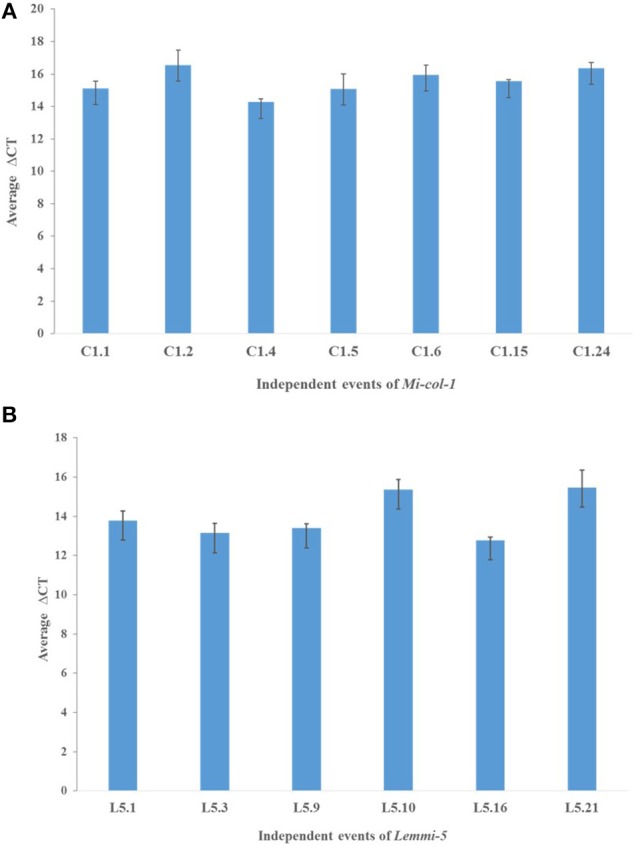
Expression analysis of **(A)** *Mi-col-1* and **(B)** *Lemmi-5* dsRNAs in their respective transgenic tomato events using qRT-PCR. *18s rRNA* (*S. lycopersicum*) gene has been used as an internal control. Each bar represents the mean ± SE of *n* = 3.

dsRNA transcript accumulation and expression was validated by qRT-PCR analysis of the selected transgenic events of *Mi-col-1* and *Lemmi-5*. An increased transcript expression was observed in all the events for *Mi-col-1* and *Lemmi-5* as compared to the wild type. However, variation in the expression was observed among the respective events. Event C1.4 showed highest expression level of *Mi-col-1* dsRNA among all the events in terms of average ΔCT values, while *Lemmi-5* dsRNA expression was found to be highest in the event L5.16 (Figure 6). These results further insured the expression of the desired dsRNAs in the transgenic events of *Mi-col-1* and *Lemmi-5*.

### Effect of host delivered RNAi on *M. incognita* infection on T_1_ tomato lines

**Figure 7:**
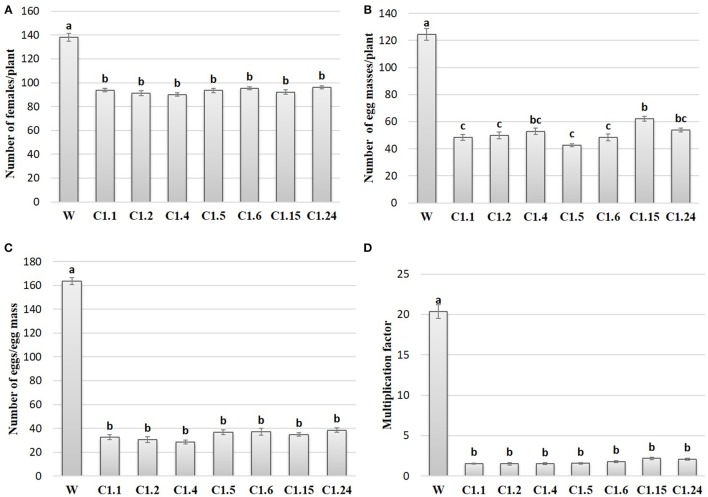
Effect of host generated RNAi of *Mi-col-1* on **(A)** relative number of females/plant; **(B)** egg masses/plant; **(C)** eggs/egg mass; and **(D)** multiplication factor in transgenic tomato events (C1.1, C1.2, C1.4, C1.5, C1.6, C1.15, and C1.24) and wild type (W) tomato plants after 35 days of inoculation. Each bar represents mean ± standard error of six different plants of each event. Bars with different letters indicate statistically significant difference between the treatments (*P* < 0.05) according to Tukey's test (Tukey's HSD).

**Figure 8:**
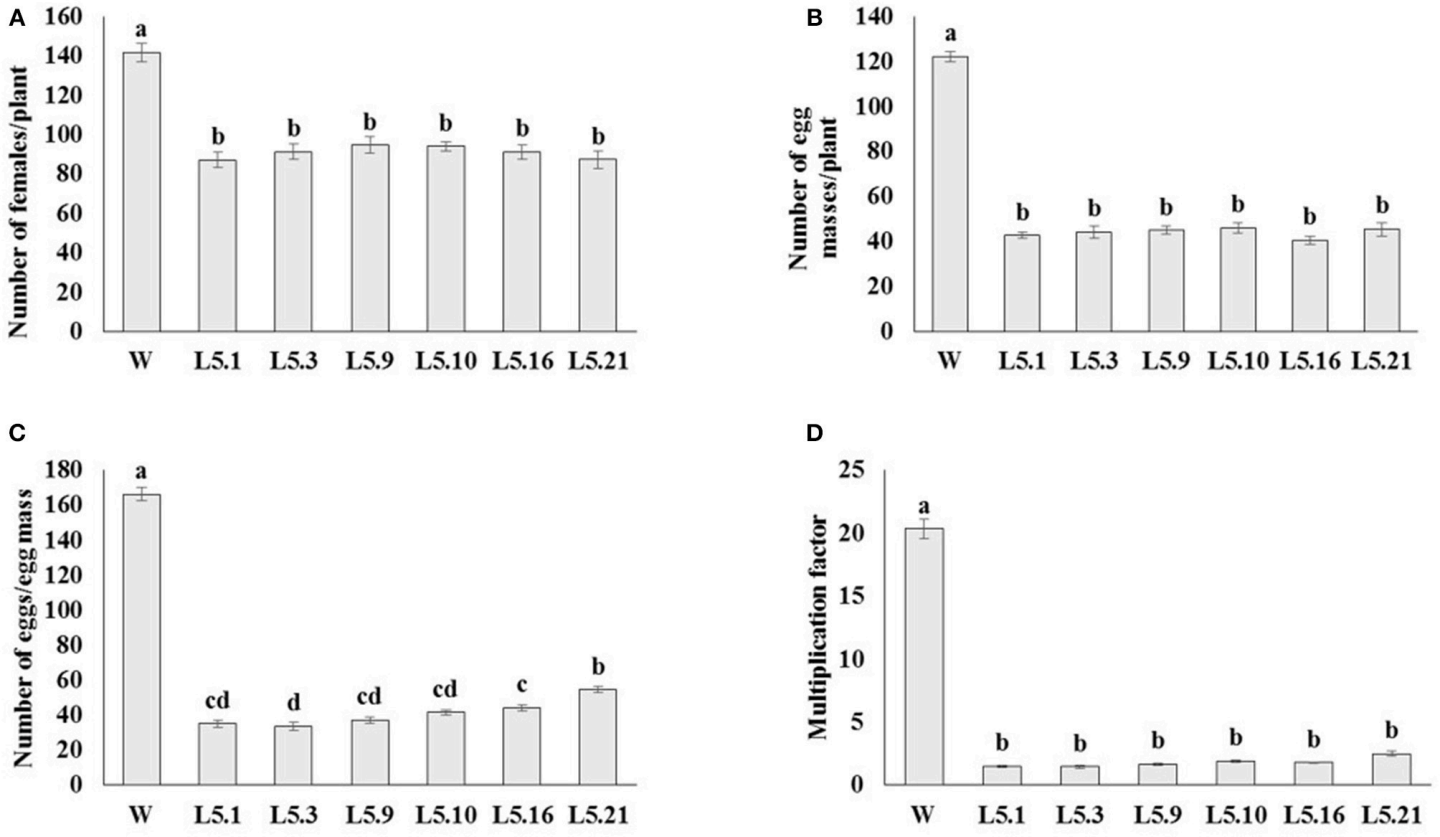
Effect of host generated RNAi of *Lemmi-5* on **(A)** relative number of females/plant, **(B)** egg masses/plant, **(C)** eggs/egg mass, and **(D)** multiplication factor in transgenic tomato events (L5.1, L5.3, L5.9, L5.10, L5.16, and L5.21) and wildtype (W) tomato plants after 35 days of inoculation. Each bar represents mean ± standard error of six different plants of each event. Bars with different letters indicate statistically significant difference between the treatments (*P* < 0.05) according to Tukey's test (Tukey's HSD).

**Figure 9:**
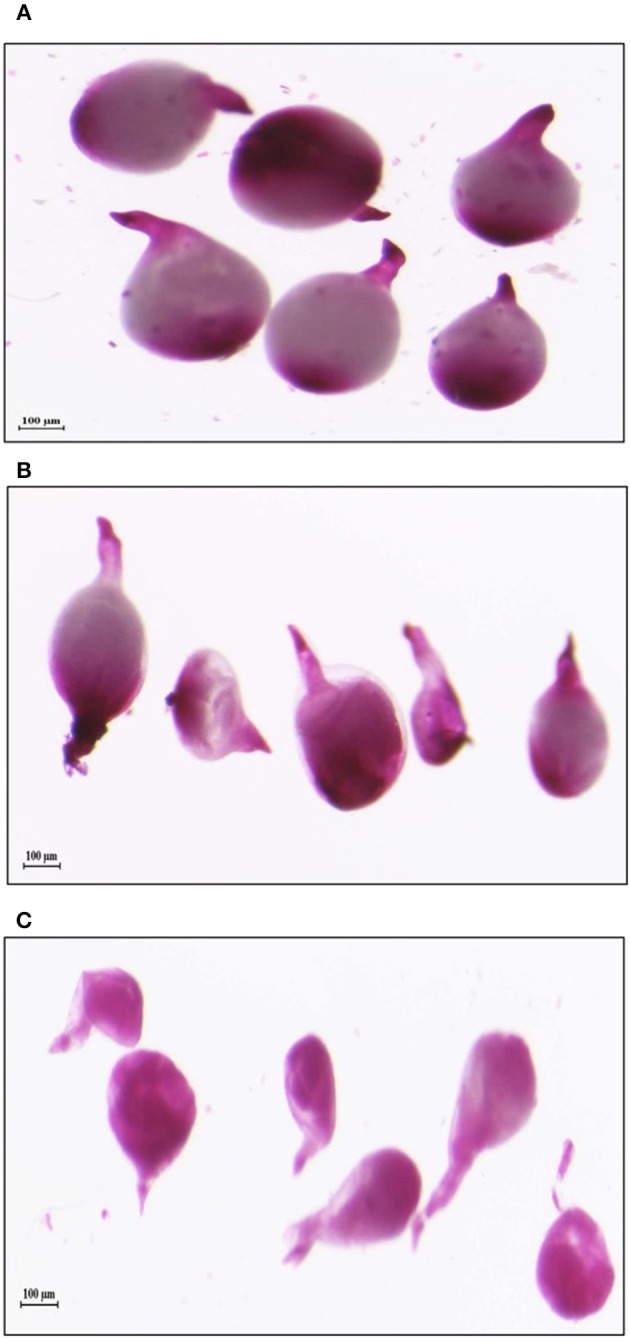
Deformation in the shape of mature females isolated from tomato plants expressing dsRNAs of **(B)** *Mi-col-1* and **(C)** *Lemmi-5* as compared to the healthy females isolated from **(A)** Wild type, 35 days post inoculation.

In order to study the effect of host delivered RNAi of *Mi-col-1* and *Lemmi-5* on *M. incognita* infection in tomato, T_1_ plants confirmed for the presence and expression of *Mi-col-1* and *Lemmi-5* were inoculated with 1,000 freshly hatched J2s/pot. T_1_ plants of seven independent events were evaluated in case of *Mi-col-1*, while six independent events were evaluated for *Lemmi-5* transgenic plants 35 days post infection. Nematode infection was scored and assessed by means of number of galls, females, egg masses, eggs per egg masses and size of adult females in T_1_ plants in comparison with wild type plants (Figures 7, 8). Reduction in the number of adult females was observed in T_1_ plants as compared to the wild type and the percentage of reduction was 30.80–35.00% for *Mi-col-1* transgenic lines and 34.15–38.54% for *Lemmi-5* transgenic lines. Furthermore, the size of the adult females was heavily distorted and deformed in both *Mi-col-1* and *Lemmi-5* transgenic lines (Figure 9). Mean area of females isolated from wild type plants was calculated as 86,580.10 μm^2^. Whereas, the mean area of females isolated from transgenic plants expressing *Mi-col-1* and *Lemmi-5* dsRNA were calculated as 50,854.10 and 37,681.40 μm^2^, respectively. Similarly, mean diameter of females isolated from wild type plants was calculated as 360.10 μm; whereas, the mean diameter of females isolated from transgenic plants expressing *Mi-col-1* and *Lemmi-5* dsRNA were calculated as 231.00 and 199.70 μm, respectively. Statistical analysis revealed significant reduction in the area and diameter of the females isolated from transgenic plants expressing *Mi-col-1* and *Lemmi-5* as compared to those isolated from wild type plants (Table 2). The fecundity of the nematodes also got hampered as indicated by the reduction in number of egg masses/plant in the range of 50.06–65.73% and 57.30–66.44% in *Mi-col-1* and *Lemmi-5* RNAi transgenic lines, respectively. The number of eggs/egg masses also reduced significantly in the range of 76.07–82.59% and 67.13–79.56% in *Mi-col-1* and *Lemmi-5* RNAi lines, respectively. Nematode multiplication factor (MF) determines successful establishment of the concerned nematode in the host plants by representing parasitic and reproductive fitness of the nematode. The multiplication factor of the nematodes in the transgenic events expressing *Mi-col-1* was calculated to be 1.51–2.17 among the events as compared to a multiplication factor of 20.39 in the wild type untransformed plants. Similarly, in transgenic events expressing *Lemmi-5* dsRNA, the multiplication factor ranged from 1.48 to 2.49 as compared to a multiplication factor of 20.35 in the wild type untransformed plants. However, No significant reduction in number of galls was observed in the transgenic events as compared to the wild type.

**Table 2:** Area and diameter of *Meloidogyne incognita* females isolated from wild type and transgenic RNAi lines.

**Treatment**	**Mean area (μm^2^)**	**Mean transformed area (sqrt)± SE**	**Mean diameter (μm)**	**Mean diameter ± SE**
Wild type	86580.10b	287.0417 ± 21.56b	360.10b	360.10 ± 11.76b
*Mi-col-1*	50854.10a	222.95 ± 11.28a	231.00a	231.00 ± 14.18a
*Lemmi-5*	37681.40a	191.6331 ± 10.31a	199.70a	199.70 ± 14.44a

**Figure 10:**
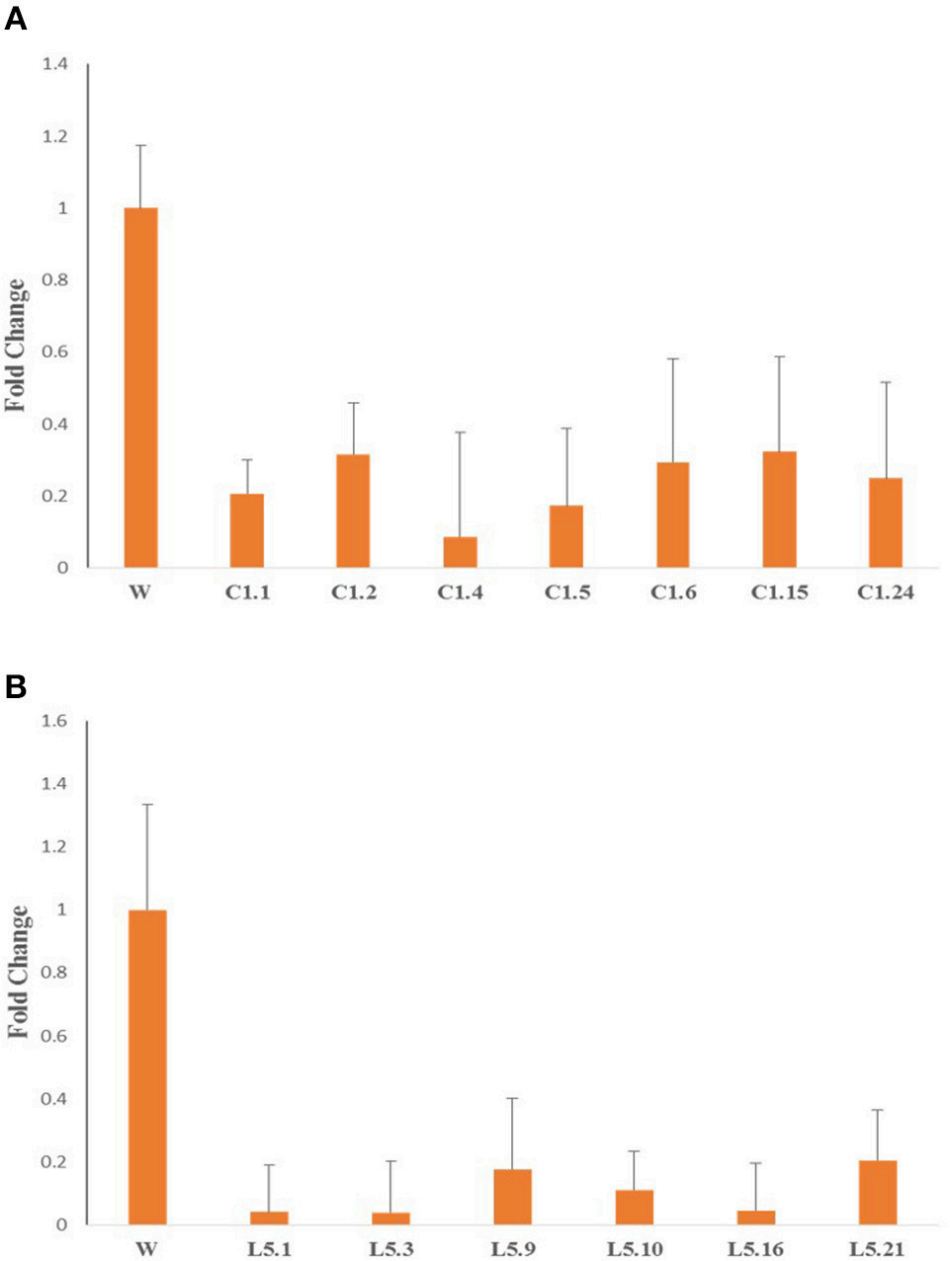
Expression of target genes **(A)** *Mi-col-1*, and **(B)** *Lemmi-5* in adult females isolated from respective transgenic tomato lines 35 days post inoculation.

qRT-PCR analysis of *Mi-col-1* and *Lemmi-5* gene expression in the females isolated from the roots of transgenic tomato events expressing the respective dsRNAs revealed significant down regulation of the target genes further indicating successful host delivered RNAi in transgenic plants. Expression levels of *Mi-col-1* was reduced in the range of 67.69–91.30% in the females isolated from tomato lines expressing *Mi-col-1* dsRNA. Similarly, the reduction in the expression levels of *Lemmi-5* was found to be in the range of 79.55–96.12% in the females extracted from the tomato transgenic lines expressing *Lemmi-5* dsRNA (Figure 10).

## Discussion

The cuticle of RKNs like *M. incognita* performs multiple functions like protection from external environment, interaction with soil and host environment, movement and locomotion and defines the shape and development of the nematode during its pre-parasitic and parasitic life cycle. About 80% of the nematode cuticle consists of collagens (Kingston, 1991). Although more than 150 cuticle collagen genes have been characterized in *C. elegans*, only a few genes have been identified in PPNs. In *M. incognita*; *Mi-col-1, Mi-col-2, Lemmi-5*, and *Mi-col-5* have been characterized and are reported to have differential expression at different stages of its life cycle (Van Der Eycken et al., 1994; Ray and Hussey, 1995; Wang et al., 1998; Banerjee et al., 2017a). Mutations in the cuticle collagen genes in *C. elegans* have resulted in morphological defects like dumpy, roller and blister phenotypes leading to larval and embryonic death (Johnstone, 2000; Page and Johnstone, 2007). RNAi of four cuticle collagen genes known as dumpy genes (*Bx-dpy-2, 4, 10*, and *11*) resulted in morphological aberrations like small dumpy body size and reduced body length in the pinewood nematode, *Bursaphelenchus xylophilus* (Wang et al., 2016). The authors demonstrated that the nematodes fed on filamentous fungus *Fusarium oxysporum* transformed with the target dsRNA constructs brought about RNAi silencing of the target dumpy genes in *B. xylophilus*. However, our study is the first report on the host generated RNAi of cuticle collagen genes in PPNs. Multiple molting of *M. incognita* during its life cycle makes the cuticle collagen genes potential targets for silencing to understand the effect and lethality of individual genes and possible chances of its effect on the overall development and parasitism of *M. incognita*.

The target genes *Mi-col-1* and *Lemmi-5* did not show similarities at the nucleotide and amino acid level. The conserved pattern of cystein residues showed that they belong to different groups of cuticle collagen genes. Phylogenetic analysis based on amino acid sequences also placed them in different clusters. However, pattern of differential expression of these genes at different life stages of the life cuticle of *M. incognita* is similar. In agreement with the earlier reports (Ray and Hussey, 1995; Wang et al., 1998), both the genes showed maximum expression in adult females followed by egg masses and J2s. However, relative folds of expression in adult females and egg masses compared to J2s were higher in case of *Lemmi-5*. Hence, despite their structural differences, both the genes appear to be involved in the cuticle development, maintenance and thickening of adult females and eggs. Unlike *Mi-col-1* and *Lemmi-5*, a recently characterized cuticle collagen gene *Mi-col-5* was reported to express more in egg masses followed by adult females and J2s (Banerjee et al., 2017a). Therefore, like *C. elegans*, the cuticle collagen formation seems to be governed by different collagen genes at different stages of the life cycle of PPNs like *M. incognita*.

Transgenic tomato plants independently expressing *Mi-col-1* and *Lemmi-5* dsRNAs were developed through *Agrobacterium* mediated transformation. The integration and inheritance of the transgenes were demonstrated by PCR and southern blotting. The expression of the dsRNAs in the respective transgenic lines analyzed through qRT-PCR also indicated stable transformation of the T-DNA harboring the dsRNA constructs of the target genes. Transgenic tomato lines were inoculated with *M. incognita* J2s to evaluate the effect of silencing of the target cuticle collagen genes through host delivered RNAi. Significant reduction in number of adult females, number of egg masses per plant and number of eggs per egg masses was observed. This indicates that both *Mi-col-1* and *Lemmi-5* are involved in the development and maintenance of the adult female cuticle which also seems to have affected the fecundity of nematodes and their ability to lay eggs as reflected by the steep reduction in the number of eggs and eggs per egg masses in the independent tomato lines expressing *Mi-col-1* and *Lemmi-5* dsRNA. Reduction in the nematode multiplication factor was also significant indicating successful repression of the nematode establishment and reproduction in the transgenic lines. Microscopic observation also revealed clear deformations in the structure of adult females feeding on these transgenic tomato lines compared to those feeding on wild type plants. The deformed structure of the adult females might have affected their reproductive potential which can be correlated to the higher expression of both the genes at the adult female stage of *M. incognita*. This could best be answered by studying the cuticle ultrastructure of these females in comparison to normally developed nematode females, but it is beyond the scope of this research work. However, it can be thought that because of improper structural development of the cuticle, the nematode female may experience non-conducive effect of the host environment, thereby affecting the female nematode's fecundity. Permeability of host molecules because of improper development of cuticle of the adult female might also have affected their reproductive potential. However, the knockdown of *Mi-col-1* and *Lemmi-5* was not able to bring about any significant reduction in gall development which indicates that these genes might not be involved in the early stages of nematode development and cuticle formation. In tomato, host generated RNAi of *Mi-Rpn7* gene of *M. incognita* also showed reduction in number of egg mass per gram of root tissue and number of eggs per gram of root tissue without any significant reduction in the number of galls (Niu et al., 2012). Similarly, gall formation was not affected by host delivered RNAi of isocitrate lyase (ICL) of *M. incognita* in tobacco, while substantial reduction in egg oviposition was reported in transgenic lines compared to wild type (Lourenço-Tessutti et al., 2015).

Expression of *Mi-col-1* and *Lemmi-5* dsRNAs in respective transgenic T_1_ plants was confirmed through real time PCR. This approach was used in earlier studies to analyze the target transcript abundance in the T_1_ plants (Papolu et al., 2013; Dutta et al., 2015). The average ΔCT values reflect the expression of target dsRNAs in the transgenic plants. Furthermore, qRT-PCR analysis of the target genes was carried out from cDNA of the adult females developed on transgenic tomato plants independently expressing *Mi-col-1* and *Lemmi-5* dsRNAs to analyse the long term effect and heritable nature of host generated RNAi. Significant reduction in the expression of the target genes was observed in adult females extracted from the transgenic plants. This indicates successful silencing of the target genes, *Mi-col-1* and *Lemmi-5* through uptake of dsRNAs/siRNAs via host generated RNAi in *M. incognita* and also suggests transmittance of the RNAi effect along subsequent moltings of *M. incognita* to the adult female stage. This systemic and heritable nature of the RNAi in PPNs was also reported in earlier studies (Fairbairn et al., 2007; Papolu et al., 2013; Dutta et al., 2015; Zhuo et al., 2016). However, it is not clear whether *M. incognita* directly ingested the dsRNAs from the host and generated the siRNAs through their own machinery or they ingested the plant processed siRNAs to bring about silencing of the target genes. Both the mechanisms are possible since RKNs are reported to ingest relatively large biomolecules (Urwin et al., 1997; Dutta et al., 2015).

Host generated RNAi of *Mi-col-1* and *Lemmi-5* has significantly hampered structure and reproduction in *M. incognita*. However, complete resistance against *M. incognita* could not be obtained. This could be due to the fact that cuticle collagen genes belong to multigene family, therefore possibility of compensatory functions of similar genes could not be denied. However, the reduction in the nematode numbers and nematode multiplication factor is comparable to the previously reported studies on host delivered RNAi to combat PPNs (reviewed by Banerjee et al., 2017b). Stacking of multiple genes for developing RNAi constructs for targeting more than one system of the nematode may be an efficient approach to achieve better degree of resistance in future studies. Application of conventional breeding for crossing two RNAi lines expressing different target leading to combined expression of both the dsRNAs also has additive effect on reduction in nematode numbers and development (Charlton et al., 2010). Gene pyramiding of PPN genes and other pathogens like virus and bacteria can also provide broad spectrum resistance against different pathogens (Walawage et al., 2013). RNAi based transgenics provide a relatively more biosafe option for developing genetically modified crops as functional proteins are not produced from the dsRNAs, which minimizes non-target effects. Moreover, humans commonly consume plants infected with virus, which produce molecules quite similar to dsRNAs or siRNAs. Therefore, these are not alien entities to human body and cells. Identification of suitable lethal target genes can be facilitated by data mining from the whole genome sequences of the key PPNs for engineering better resistance. Use of tissue specific and nematode induced promoters may address the biosafety concerns better by limiting the dsRNA expression in response to specific nematodes and in specific plant tissues.

## Author contributions

AS, SG, PJ, KS, and SB conceived and designed the experiments. SB performed the experiments. SB and BG analyzed the data. SB wrote the manuscript. AS, KS, PJ, and SG critically revised the manuscript. All authors read and approved the final manuscript.

### Conflict of interest statement

The authors declare that the research was conducted in the absence of any commercial or financial relationships that could be construed as a potential conflict of interest.
