# Supplementary material for: Host Delivered RNAi of Two Cuticle Collagen Genes, Mi-col-1 and Lemmi-5 Hampers Structure and Fecundity in Meloidogyne incognita
Source: Front Plant Sci. 2018 Jan 22;8:2266. doi: 10.3389/fpls.2017.02266 (PMC5786853; doi:10.3389/fpls.2017.02266)
Supplement: Supplementary file 1 [file Table1.DOCX]

**TABLE S1** Primary structural properties of *Mi-col-1* and *Lemmi-5* amino acid sequences.

| **SN** | **Parameter** | **Theoretical prediction** | |
| --- | --- | --- | --- |
|  |  | ***Mi-col-1*** | ***Lemmi-5*** |
| 1 | Molecular weight (kDa) | 29.47 | 30.58 |
| 2 | Isoelectric point | 8.01 | 9.87 |
| 3 | Total no. of negatively charged residues (Asp+Glu) | 16 | 10 |
| 4 | Total no. of positively charged residues (Arg+Lys) | 18 | 28 |
| 5 | Extinction coefficient (M^-1^ cm^-1^, at 280 nm) | 15065 | 12085 |
| 6 | Instability index | 51.80 | 58.86 |
| 7 | Aliphatic index | 35.44 | 48.03 |
| 8 | Grand average of hydropathicity (GRAVY) | -0.683 | -0.714 |
