# Supplementary material for: Host Delivered RNAi of Two Cuticle Collagen Genes, Mi-col-1 and Lemmi-5 Hampers Structure and Fecundity in Meloidogyne incognita
Source: Front Plant Sci. 2018 Jan 22;8:2266. doi: 10.3389/fpls.2017.02266 (PMC5786853; doi:10.3389/fpls.2017.02266)
Supplement: Supplementary file 2 [file Presentation1.PDF]

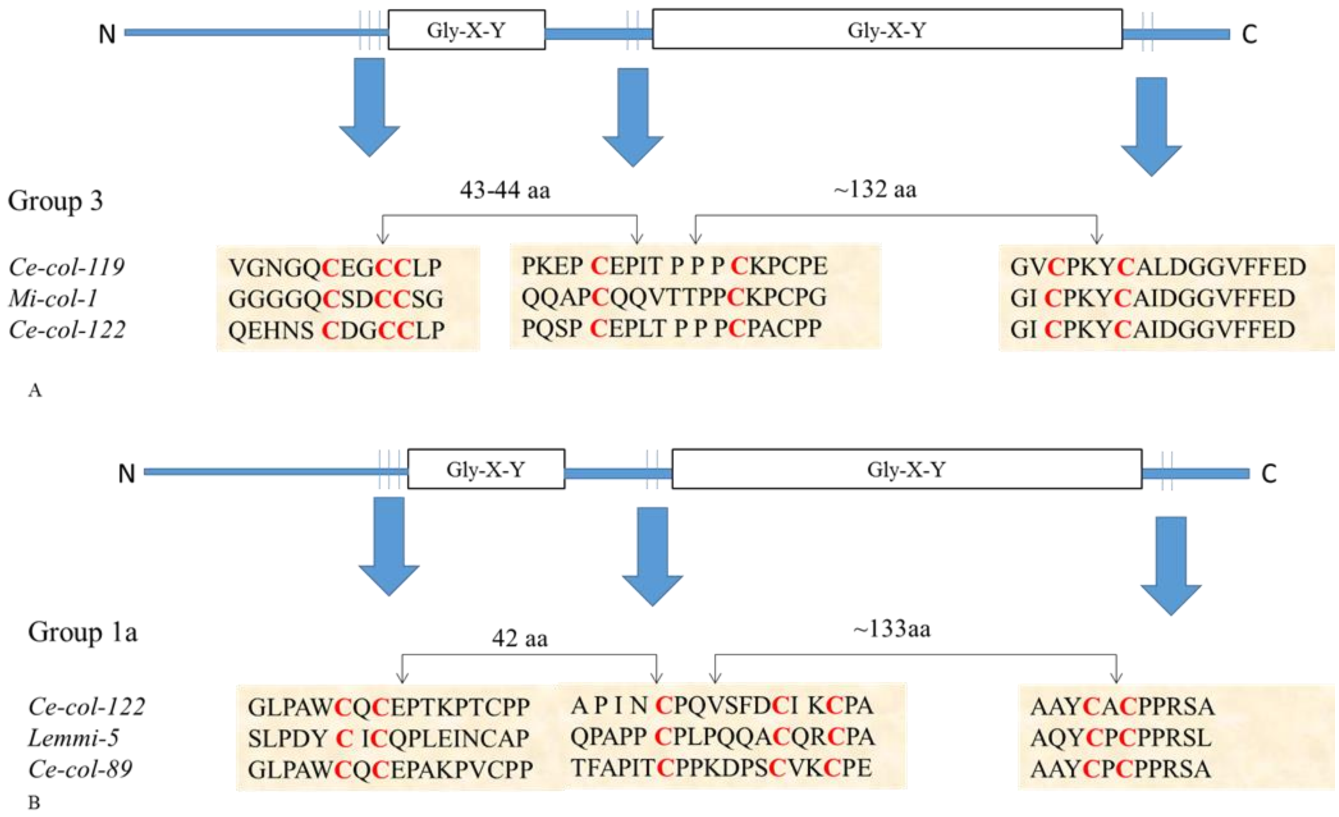

Figure S1: Conserved pattern of cysteine residues classifies (A) *Mi-col-1* in group 3 and (B) *Lemmi-5* in group 1a of cuticular collagen genes of nematodes. The positions of Gly-X-Y domains along three cysteine containing domains are represented here. This system of classification of cuticular collagen genes was proposed by Johnstone, 2000 on the basis of conserved patterns of cysteine residues.

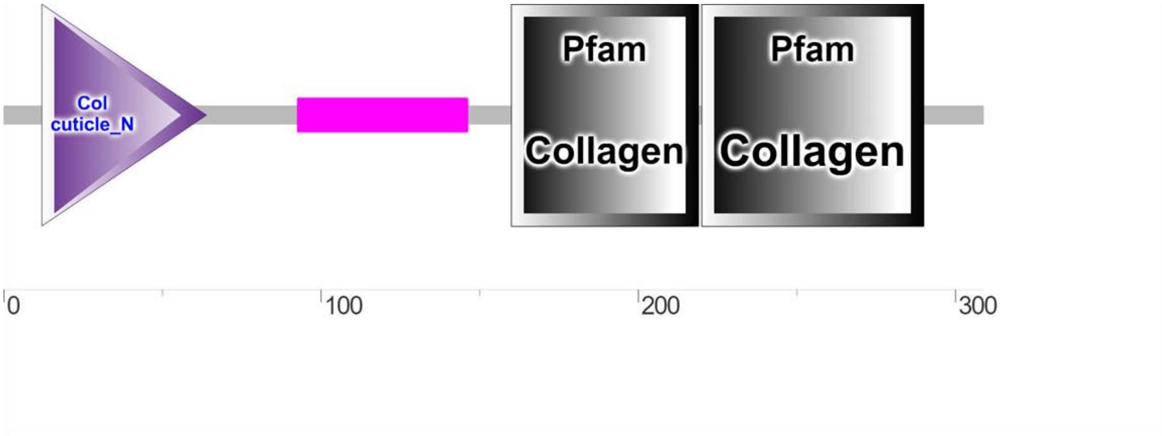

A

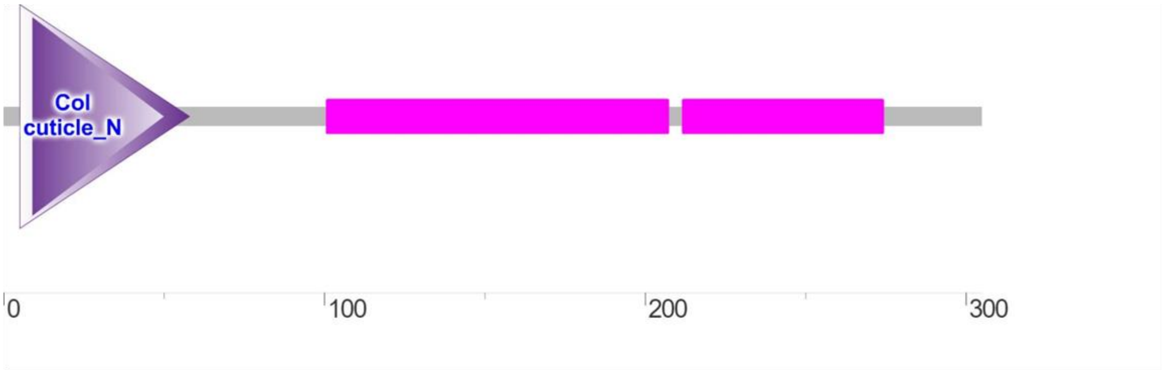

B

Figure S2: Domain prediction for (A) *Mi-col-1* and (B) *Lemmi-5* by SMART. Red segments represent predicted low complexity regions.

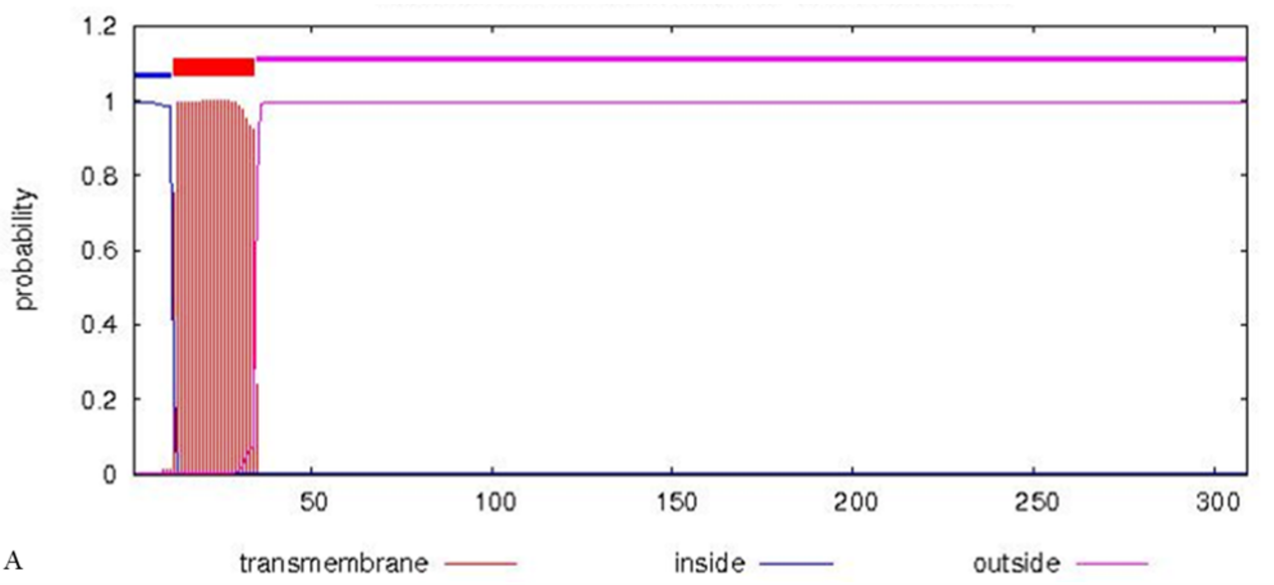

A

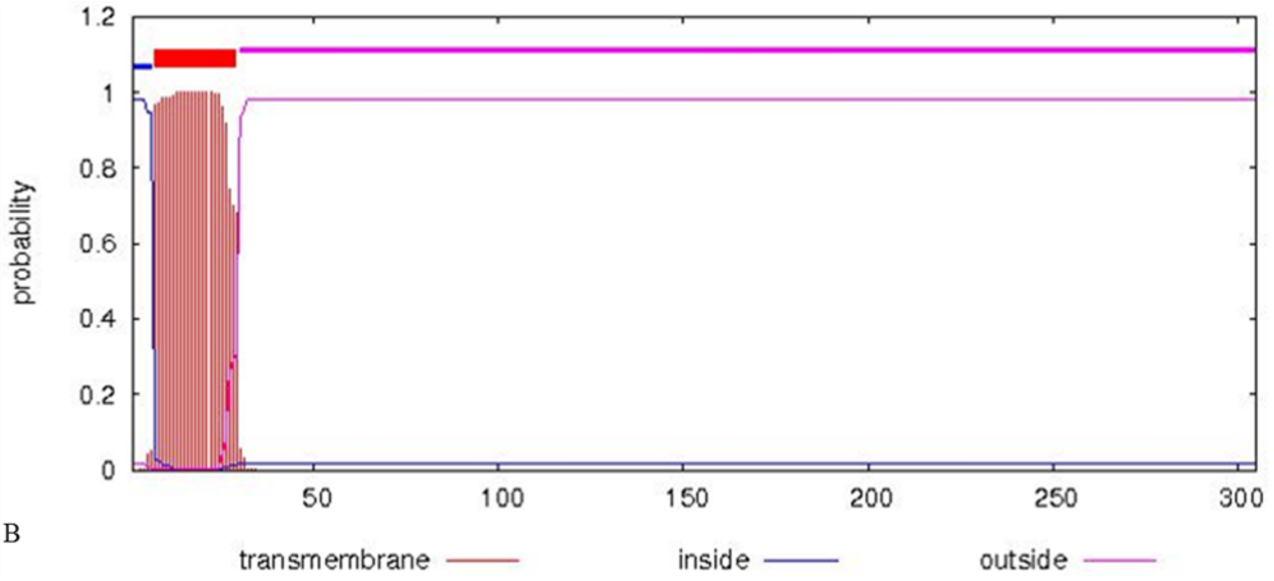

B

Figure S3: TMHMM predictions for (A) *Mi-col-1* (B) *Lemmi-5*. Blue, red and pink colors represent the portions of the amino acids inside, in the transmembrane helix and outside the cell respectively
